# Midwife-led care versus obstetrician-led care for childbearing women in early labor: A systematic review

**DOI:** 10.18332/ejm/218434

**Published:** 2026-06-15

**Authors:** Antonia N. Mueller, Piroska Zsindely, Vanora Hundley, Rahel Naef, Lauren Clack, Susanne Grylka-Baeschlin

**Affiliations:** 1 Research Institute of Midwifery and Reproductive Health, School of Health Sciences, ZHAW Zurich University of Applied Sciences, Winterthur, Switzerland; 2 Faculty of Medicine, University of Zurich, Zurich, Switzerland; 3 Centre for Midwifery & Women's Health, Bournemouth University, Bournemouth, United Kingdom; 4 Implementation Science in Nursing, Faculty of Medicine, University of Zurich & Centre for Clinical Nursing Science, University Hospital Zurich, Zurich, Switzerland; 5 Institute for Implementation Science in Health Care, Faculty of Medicine, University of Zurich, Zurich, Switzerland; 6 Department of Infectious Diseases and Hospital Epidemiology, University Hospital Zurich, Zurich, Switzerland

**Keywords:** care models, midwifery care, latent phase of labor, labor onset, medical interventions

## Abstract

**INTRODUCTION:**

Women admitted to the hospital early in labor face an increased intrapartum intervention rate, possibly resulting in negative obstetric outcomes. It is well documented that women receiving midwife-led care receive fewer unnecessary medical interventions. However, the impact of midwife-led care during early labor remains poorly understood. The aim of this study was to evaluate the effect of midwife-led care compared to obstetrician-led care regarding medical interventions during early labor.

**METHODS:**

A systematic review of literature published until June 2024 was performed in PubMed, CINAHL Complete, Web of Science Core Collection, and the Cochrane Library following Cochrane guidelines. PICO criteria included the keywords pregnant women, midwife-led care, obstetrician-led care and medical interventions during early labor. Quality was assessed using the RoB 2-tool and the ROBINS-I-tool. Data were extracted by using a purposively designed extraction template and then analyzed descriptively.

**RESULTS:**

Of 1057 identified studies, four studies were eligible and included in this review, including two randomized controlled trials and two observational studies. The results regarding birth mode are not entirely clear. Most studies reported that women who receive midwife-led care during the early stages of labor are more likely to have a vaginal birth and less likely to require a cesarean section. However, one study could not find a statistically significant difference regarding birth mode and care model received in early labor. Another study showed increased use of labor augmentation among women receiving obstetrician-led care.

**CONCLUSIONS:**

There remains a lack of knowledge about the role of midwife-led care during early labor and its impact on early labor interventions and subsequent birth outcomes. More attention should be focused on early labor care to improve outcomes for laboring women and their partners. Recognizing the potential benefits of midwife-led care during this phase could lead to initiatives aimed at promoting such care across various settings.

## INTRODUCTION

Care in early labor can influence the further course of childbirth^1^. Early labor, also referred to as the latent phase of labor, describes the beginning of the first stage of labor, and there is much debate within the literature regarding its definition^2,3^. To date, there is no standardized or well-defined approach for testing the onset of early labor^3^. However, various symptoms such as contractions, gastrointestinal symptoms, exhaustion, or cervical dilation (1–6 cm) are described^2^.The lack of clear criteria for diagnosis for this phase and the heterogeneous definition of early labor can create difficulties in managing it for both pregnant women and healthcare professionals^4^.

Women in early labor frequently benefit from substantial emotional support and reassurance. Often these women seek hospital admission to receive advice on how to cope with early labor and to gain a feeling of being safe^5^. Yet, for some women, the need for hospital admission coincides with medical interventions. Some women may be comforted by knowing their cervical dilation, and some may favor medical pain management, such as epidural anesthesia during early labor, to be able to cope with the pain^4^. Although medical interventions such as vaginal examinations and hemorrhage prevention undoubtedly have a positive impact on outcomes and decrease maternal and fatal mortality rates, childbirth in Western countries has become overmedicalized^6,7^. This phenomenon paradoxically promotes the occurrence of various negative health outcomes for the mother and child^6^ and potentially jeopardizes women-centered maternity care. Evidence shows that diverse medical interventions during childbirth are often applied without the receipt of informed consent^6,8^. In particular, women who are admitted to hospital early in labor are more likely to undergo intrapartum interventions associated with negative outcomes such as amnionitis, operative birth mode, longer hospital stay, fatal distress, lower APGAR scores (assessing neonatal status including appearance, pulse, grimace, activity, and respiration), and higher risk of neonatal resuscitation^1,9-11^. Health-related costs are further arguments for late hospital admission in labor since medical interventions such as epidural analgesia or cesarean section have a great impact on increased cost development^11^. As a consequence, midwives often act as gatekeepers for women in early labor and advise them to stay at home^12^. This might not be in the interest of all women, as some women favor early hospital admission^12^ and therefore may result in lower satisfaction with care^13^. There is a need to balance early hospital admission with care in early labor. According to Coates et al.^14^ and Reime et al.^15^, obstetricians are more likely to agree on the administration of medical interventions during early labor than midwives and also to view the intense pain of early labor as requiring hospital admission. On the other hand, various measures such as home visits or early labor lounges have gained interest in managing early labor to support later admission to the labor ward^1,16^.

The organization of maternity care models varies widely and lacks a clear definition^17,18^. Especially when focusing on midwife-led care, there seem to be different conceptions. Sandall et al.^19^ describe midwife-led care models as those as those in which the midwife takes the lead in providing maternity care to pregnant women. In the latest review, Sandall et al.^20^ focus more on midwifery continuity of care models where midwives provide care throughout pregnancy, childbirth, and the postpartum period. But midwife-led care does not necessarily include a continuum of care. This means that the midwife may be the lead health professional only during a specific period from pregnancy to the postpartum period^17,18^. Apart from midwife-led care models, other models of care exist. These include the medical-led care model, also frequently described as standard care, but in this article referred to as obstetrician-led care, where an obstetrician provides antenatal care and takes responsibility for labor and birth as well^20^. Continuity of carer is often lacking within obstetrician-led care since antenatal care may be provided by private obstetricians, while care during labor is provided by hospital staff^7,21^. Shared models of care are those where medical doctors and midwives share their responsibility for the care provided^20,22^. In 2014 a Lancet series was published that highlighted the contribution of midwifery care towards better maternal and newborn health^8,23^. Midwifery care was shown to significantly promote spontaneous vaginal births, reduce operative deliveries or medical interventions such as episiotomy, and support a higher level of satisfaction with maternity care^20^. Due to the known benefits of midwifery care, several countries, such as the UK, the Netherlands, Australia, New Zealand, and Iceland, have implemented midwife-led care models in hospitals^21,24^. Despite the variety of research regarding different models of care and their contribution to childbirth interventions and women’s satisfaction with care received during childbirth in general^7^, little is published about early labor. There is a lack of knowledge regarding the impact of diverse models of care provided during the vulnerable stage of early labor^1,25^.

This systematic review aimed to evaluate the effect of midwife-led care compared to obstetrician-led care regarding medical interventions during early labor in childbearing women.

## METHODS

A systematic review following the guidance of the Cochrane Handbook for Systematic Reviews by Higgins and Thomas^26^ and the Preferred Reporting Items for Systematic Reviews and Meta-Analyses (PRISMA) guidelines for reporting^27^ was conducted. To allow transparency, a study protocol was published on PROSPERO (ID: CRD42022376176).

### Search strategy

The search strategy was developed according to the protocol by Hirt and Neuhauser^28^ and comprised four different databases, including PubMed, CINAHL Complete, Web of Science Core Collection, and the Cochrane Library. These databases have been selected due to their suitability for the researched subject, but also because of their adequacy for a sensitive literature search. PICO criteria were defined using pregnant women as the population, midwife-led care as the intervention, obstetrician-led care as the control, medical interventions during early labor, and satisfaction with early labor care as the outcome.

To retrieve all relevant research articles on the topic, various synonyms and database-related subject headings were used and combined using the Boolean operators AND and OR. The development of the search string was checked by S-GB (second researcher) and PZ (third researcher), which led to the implementation of more gender-neutral keywords specifically for the component population, such as ‘pregnant people’ and other synonyms for ‘obstetrician-led care’. An example of the search strategy on PubMed is shown in Supplementary file Annex 1 corresponding to PubMed No. 11. The full search strategies can be found in Supplementary file Annex 1. A first literature search showed that the outcome keywords were too narrow. Yet, deleting the focus on early labor resulted in too many hits. According to Higgins and Thomas^26^ the outcome should be left out of the search string to better allow for a sensitive search strategy. Therefore, a second search was performed without the outcome variable (Supplementary file Annex 1 corresponding to PubMed No. 12). To guarantee the accuracy of this procedure, the search strategy was discussed with a librarian specialized in health care database searching. The Cochrane guidelines recommend a search on MEDLINE. Since citations by MEDLINE are included in PubMed^28^, which offers additional citations, this was considered sufficient. The search string was checked regarding keywords, wildcards, and operators, and rated as accurate. A further search strategy meeting with S-GB led to the finalization of the search strategy. The final search was performed on 12 January 2023, and updated on 4 June 2024.

### Eligibility

All original, quantitative studies of experimental and quasi-experimental or observational design were considered for this review due to the possible natural exposure of the intervention. Since this review aimed to understand the differences between care models, only comparative designs were included. The articles did not have any restrictions on publication dates, yet were only considered if the full text was available in German, English, French, Spanish, or Italian.

*Population*

The review focused on pregnant women during early labor with spontaneous onset of labor. Therefore, articles focusing on a population with medical induction of labor or elective cesarean section were excluded. Any other mode of birth was not restricted.

*Intervention and comparison*

In some countries, midwife-led care refers to a standard model of care for women and their children; therefore, clarification regarding intervention and comparison was essential. Midwife-led care was defined as a model of care in which the midwife is the lead health professional for women in maternity care^21^. Obstetrician-led care, as a comparator, also built upon the definitions of Sandall et al.^19^ where obstetricians or any medical doctors act as the lead provider of maternity care. Early labor was referred to as either before 4 cm cervical dilatation^29^ or as care received at the onset of labor^30-32^.

*Primary and secondary outcomes*

The primary and secondary outcomes are listed in Table 1.

**Table 1:** Outcomes of the systematic review

Primary outcomes	Secondary outcomes
Labor augmentation including pharmacological, mechanical, or alternative options Pharmacological analgesia Tocolysis	Satisfaction with early labor care Early labor duration Mode of birth according to care received during early labor

### Screening and selection process

Screening was performed in accordance with the procedures proposed by Peters et al.^33^. Articles retrieved from the search process were imported into Zotero reference management software. In a second step, the references were transferred to Covidence, a systematic review management tool that allows several researchers to screen and assess articles independently^34^. Duplicates were removed using the aforementioned management tool. Following this, three researchers were engaged in screening, selecting, and extracting the data.

Ambiguities in the definition of early labor and obstetrician-led care were resolved by a meeting between the three researchers, which was held at the beginning of the screening and selection process to find consensus on how to handle studies without a clear definition of the care model used and without a clear differentiation of the different stages of labor. Studies that did not clearly focus either on interventions during early labor and studies that researched birth mode, yet without classifying the aimed model of care during early labor, were regarded as ineligible.

### Quality assessment

The quality of the studies was assessed using the RoB 2 tool for randomized controlled trials^35^ and the ROBINS-I tool for observational studies^36^, and was performed by two researchers independently (ANM and SG-B/PZ). Both tools are validated and standardized, including various components such as randomization process, deviation of the intended interventions, measurement of data, control of confounding factors, and selection of participants. Overall ratings were derived from individual scores. To assess quality, information was retrieved from the articles. For two studies, study protocols were accessible, which helped the assessment^30,32^. A study protocol for Jackson et al.^29^ could not be found, and for Gu et al.^31^ was only accessible in Chinese.

### Data extraction

Data were extracted separately by two researchers (ANM and SG-B/PZ) within Covidence by using a purposively designed extraction template. This included information on the study, methods used, population and setting, description of the interventions, controls, and outcomes.

### Data analysis

Data were analyzed descriptively. Due to the high heterogeneity of the extracted data, meta-analysis was not possible^26^. This was approved by a statistics consultant. The studies differed regarding design (two RCTs and two observational studies) and regarding effect sizes. They were also heterogeneous regarding intervention and outcomes. In addition to this heterogeneity, the small number of included studies supported the low quality of a potential meta-analysis.

## RESULTS

**Figure 1:**
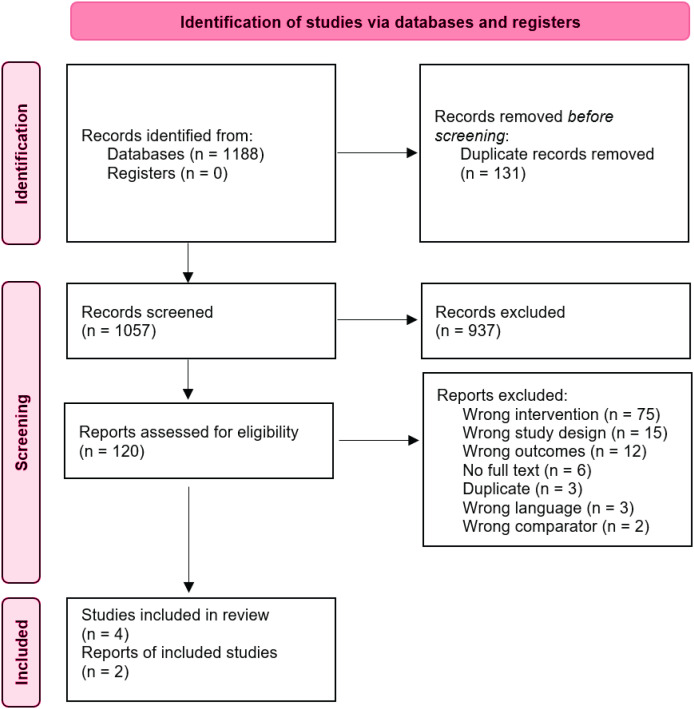
PRISMA flowchart of identified studies of the systematic review

A total of 1057 titles and abstracts were screened after duplicate removal, leading to 120 full texts for closer examination (Figure 1). Reasons for further exclusion of studies were mainly due to the intervention not being midwife-led care (n=75) or because the outcome did not separately focus on early labor (n=12). Further studies were excluded due to wrong study design (n=15), wrong language (n=3), wrong comparator (n=2), or because full text was not available even after contacting the authors (n=6). This resulted in four included studies in this review.

### Study characteristics

The included studies included two cohort studies^29,30^ and two randomized controlled trials (RCT)^31,32^. The studies were conducted between 2003 and 2015 in the United States, China, the Netherlands, and Australia. A description of the included studies can be found in Table 2. All study participants had a singleton pregnancy, were at low obstetric risk, and were booked at the participating study centres^29-32^.

**Table 2:** Description of included studies in the systematic review

*Study details*	*Jackson et al.^29^*	*Gu et al.^31^*	*Tracy et al.^32^*	*de Jonge et al.^30^*
**Title**	Impact of collaborative management and early admission in labor on method of delivery	The effectiveness of a Chinese midwives‘ antenatal clinic service on childbirth outcomes for primiparae: a randomized controlled trial	Caseload midwifery care versus standard maternity care for women of any risk: M@NGO, a randomized controlled trial	Severe Adverse Maternal Outcomes among Women in Midwife-Led versus Obstetrician-Led Care at the Onset of Labor in the Netherlands: A Nationwide Cohort Study
**Journal**	Journal of Obstetric, Gynecologic, & Neonatal Nursing	International Journal of Nursing Studies	Lancet	PLOS ONE
**Setting**	United States	China	Australia	Netherlands
**Study design**	Cohort study	Randomized controlled trial	Randomized controlled trial	Cohort study
**Population description**	Women with a singleton pregnancy at low risk with a fetus in cephalic presentation and spontaneous labor onset at term.	Primiparous women with low risk and singleton pregnancy without planned cesarean section	Women aged 18 years old with a singleton pregnancy without planned cesarean section.	Women with a singleton pregnancy between 37 and 42 weeks gestation without a previous cesarean section, with spontaneous onset of labor and a child in cephalic presentation.
**Number of participants Intervention (Control)**	1413 (783)	53 (53)	871 (877)	170439 (53300)
**Description of intervention**	Obstetricians and midwives who worked as a team. Midwives managed labor for low-risk women in a birthing center. If women were of any risk, childbirth was managed collaboratively within the hospital setting.	The midwife usually focused on antenatal checkups, consultation, making birth plans, parent education, and collaborated with obstetricians and other health professionals as necessary. The midwife would be on call for the woman’s labor and birth. Each woman had a chance of having continuous one-toone care from the onset of labor to 2 h postpartum.	Caseload midwifery care where women receive antenatal, intrapartum and postpartum care in a hospital setting and in the community from a named caseload midwife who works within a small group. When urgent assistance was needed in hospital it was provided by the rostered medical staff. In addition to providing care throughout pregnancy, labor, and birth, the named caseload midwife (or a backup midwife if the named midwife was unavailable) attended the woman in hospital to provide postnatal care and advice until discharge.	Births that started in midwife-led care
**Description of comparator**	Obstetricians and obstetric residents provided perinatal care in the hospital setting.	Women allocated to the control group were given the routine obstetrician-led antenatal care. When women came into the hospital for labor and birth, they would be cared for by whichever midwives and obstetricians were rostered for duty.	Obstetrician-led care at both hospitals included shared care from a general practitioner and hospital midwives. Standard hospital care was provided through antenatal clinics, labor wards, and postnatal wards, where care is provided by rostered medical and midwifery staff. In obstetrician-led care, women could potentially see a different midwife for every visit.	Births that started in obstetrician-led care
**Primary outcome**	Mode of birth	Childbirth outcomes, women’s psychological state and satisfaction	Mode of birth; epidural analgesia; APGAR score; admission to NICU; preterm birth	Severe acute maternal morbidity
**Secondary outcome**		Blood loss 2 h pp; APGAR score	Antenatal admission to hospital; induction or augmentation of labor; perineal status after birth; blood loss after birth; gestational ages and birthweights of the infants; breastfeeding at hospital discharge, 6 weeks and 6 months postnatally; and perinatal and maternal mortality	Postpartum hemorrhage; manual removal of placenta

APGAR: Appearance, Pulse, Grimace, Activity, Respiration. NICU: Neonatal Intensive Care Unit.

The four studies differed slightly in terms of the model of care. Tracy et al.^32^ focused on caseload midwifery, where a group of midwives is fully responsible for the antenatal, intrapartum and postpartum care and provides continuity in care^32^. Gu et al.^31^ described the intervention as a group of midwives that provided care during the antenatal period and were then responsible for the care provided during childbirth within the hospital setting^31^. In both RCTs, obstetrician-led care was described as shared care by rostered medical and midwifery staff onsite^31,32^. In de Jonge et al.^30^, data from the national perinatal database where women were reported to have received midwife-led care at the onset of labor were compared with data where care at the onset of labor was obstetrician-led^30^. Jackson et al.^29^ investigated perinatal outcomes among women receiving care shared within a team by midwives and obstetricians. In this study, midwives were the lead care providers, while obstetricians provided perinatal care at one of the participating centers. Notably, these four care models were already applied during early labor^29-32^. All studies documented results regarding birth mode according to the care model received during early labor. Only one study provided a clear definition of early labor – Jackson et al.^29^ reported this as care received before 4 cm of cervical dilatation. The study by de Jonge et al.^30^ was the only one that reported early labor interventions, with a specific focus on labor augmentation.

### Quality assessment

**Figure 2:**
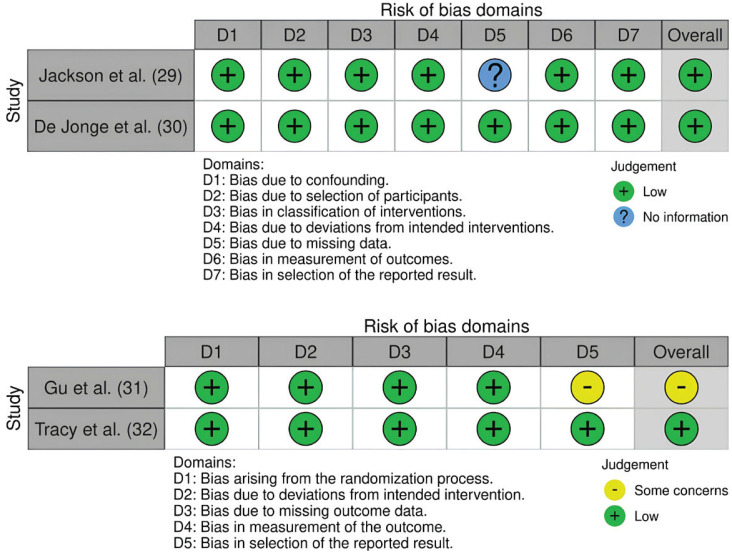
Quality assessment using the RoB2-tool for randomized controlled trials (Gu et al.^31^ and Tracy et al.^32^) and the ROBINS-I-tool for observational studies (Jackson et al.^29^ and de Jonge et al.^30^)

The overall risk of bias for the study by Tracy et al.^32^ was considered low. Possible bias in reporting results due to an unavailable pre-specified analysis plan raised some concerns regarding the overall quality of the study by Gu et al.^31^. Although there was a lack of information on missing data in the study by Jackson et al.^29^, after discussion with all three researchers, the overall risk of bias was found to be low. Also, the overall risk of bias for the study by de Jonge et al.^30^ was assessed to be low. A summary of the assessed risks of bias is shown in Figure 2.

### Labor augmentation

In de Jonge et al.^30^, labor was augmented in 27.4% of nulliparous women who were in midwife-led care at the onset of labor and in 43.5% nulliparous women who were in obstetrician-led care at the onset of labor. With regard to multiparous women, labor augmentation was performed in 6.1% of women within midwife-led care and in 23.0% in women within obstetrician-led care.

### Mode of birth according to care received during early labor

Birth mode was documented by all the included studies (Table 3). Tracy et al.^32^ stated that spontaneous birth occurred in 487 women (56%) in midwife-led care and 454 women (52%) in obstetrician-led care (OR=1.18; 95% CI: 0.98–1.43, p=0.08). Instrumental birth was documented in 172 women (20%) in midwife-led care and 171 women (19%) in obstetrician-led care (OR=1.02; 95% CI: 0.80–1.29, p=0.90). With regard to cesarean section, similar results occurred between the two groups with 182 (21%) in the intervention group and 204 (23%) in the control group (OR=0.88; 95% CI: 0.70–1.10, p=0.26). Thus, the mode of birth was not statistically significantly different between the two groups^32^. In contrast, the RCT by Gu et al.^31^ found a significant increase in vaginal births (66.04% vs 43.40%, risk difference RD=22.64%; 95% CI: 3.69–41.60) and a decrease in cesarean section (33.96% vs 56.60%, RD= -22.64%; 95% CI: -41.60 – -3.69, p=0.019) among women in midwife-led care versus obstetrician-led care. Also, Jackson et al.^29^ showed higher rates of spontaneous birth for women in the midwife-led care model and higher risks for vaginal operative childbirth for women in the obstetrician-led care group. The risk of having a cesarean section was only significantly lower in multiparous women with previous operative childbirth, but not for nulliparous women or multiparous women without previous cesarean section. Additionally, the descriptive analysis of de Jonge et al.^30^ showed higher rates of operative childbirth, including vaginal operative and cesarean section, for women receiving obstetrician-led care.

**Table 3:** Outcome measures of included studies in the systematic review

Authors Year	Statistical measurement	Intervention group	Control group	Effect size
**Jackson et al.^29^ 2003**	General linear model with binominal distribution and identity link to obtain adjusted risk differences and likelihood ratio or Wald 95% confidence limits	Mode of birth % Spontaneous Nullipara: 61.0 Multipara no previous CS: 90.1 Multipara with previous CS: 68.4 Vaginal operative Nullipara: 21.9 Multipara no previous CS: 4.3 Operative/cesarean section Nullipara: 17.1 Multipara no previous CS: 5.7 Multipara with previous CS: 31.6	Mode of birth (%) Spontaneous Nullipara: 45.6 Multipara no previous CS: 76.1 Multipara with previous CS: 38.2 Vaginal operative Nullipara: 34.2 Multipara no previous CS: 18.9 Operative / cesarean section Nullipara: 20.2 Multipara no previous CS: 5.0 Multipara with CS 61.8	Mode of birth ARD (95% CI) Spontaneous Nullipara: -15.4 (-18.9 – -11.0) Multipara no previous CS: -10.5 (-11.4 – -8.6) Multipara with previous CS: -30.2 (-56.7 – -3.7) Vaginal operative Nullipara: 9.1 (7.8–12.3) Multipara no previous CS: 12.0 (10.6–12.8) Operative/cesarean section Nullipara: 6.9 (-11.7–8.7) Multipara no previous CS: -0.7 (-1.7–0.4) Multipara with previous CS: 30.2 (3.7–56.7)
**Gu et al.^31 2013^**	T-test for mean values and Pearson’s chisquared test for categorical data	Mode of birth n (%) Spontaneous and vaginal operative 35 (66.04) Operative/cesarean section 18 (33.69)	Mode of birth n (%) Spontaneous and vaginal operative 23 (43.40) Operative / cesarean section 30 (56.60)	Mode of birth RD (95% CI) Spontaneous and vaginal operative 22.64% (3.69–41.60) Operative/cesarean section -22.64% (-41.60 – -3.69), p=0.019
**Tracy et al.^32 2013^**	Univariate logistic regression for odds ratios with 95% CIs and Pearson χ^2^ tests for p-values; for non-normally distributed data non-parametric bootstrap percentile CIs were used	Mode of birth n (%) Spontaneous 487 (56) Vaginal operative 172 (20) Operative/cesarean section 183 (21)	Mode of birth n (%) Spontaneous 454 (52) Vaginal operative 171 (19) Operative/cesarean section 204 (23)	Mode of birth OR (95% CI) Spontaneous 1.18 (0.98–1.43), p=0.08 Vaginal operative 1.02 (0.80–1.29), p=0.90 Operative/cesarean section 0.88 (0.70–1.10), p=0.26
**de Jonge et al.^30 2015^**	Multivariable logistic regression for adjusted odds ratio with 95% CI	Augmentation of labor n (%) Nulliparous: 20943 (27.4) Multiparous: 5739 (6.1) Mode of birth n (%) Operative including cesarean section and vacuum/forceps delivery Nulliparous: 18366 (24.0) Multiparous: 2049 (2.2)	Augmentation of labor n (%) Nulliparous: 10544 (43.5) Multiparous: 6684 (23.0) Mode of birth n (%) Operative including cesarean section and vacuum/forceps delivery Nulliparous: 8841 (36.5) Multiparous: 3347 (11.5)	Not assessed for labor augmentation and birth mode.

CS: cesarean section. ARD: adjusted risk difference. p-value: studies reporting p set at 0.05.

Two studies created comparison groups according to birth mode and parity. Jackson et al.^29^ investigated birth mode in nulliparous women, multiparous women without a history of cesarean section, and multiparous women with a history of cesarean section and received model of care before 4 cm cervical dilatation. Among nulliparous women, 61.0% gave birth spontaneously in the midwife-led group compared to 45.6% in the obstetrician-led group (adjusted risk difference, ARD= -15.4; 95% CI: -18.9 – -11.0). In multiparous women without prior cesarean section, the numbers for spontaneous birth were 90.1% for the intervention group and 76.1% for control (ARD= -10.5; 95% CI: -11.4 – -8.6) and for multiparous women with prior cesarean section 68.4% vs 38.2% (ARD= -30.2; 95% CI: -56.7 – -3.7). Within the intervention group, 21.9% of nulliparous women had an assisted delivery compared to 34.2% in the control group (ARD=9.1; 95% CI: 7.8–12.3). Cesarean section was documented for 17.1% of nulliparous women in the intervention group and 20.2% in the control group (ARD=6.9; 95% CI: -11.7–8.7). Assisted delivery for multiparous women without prior cesarean section was 4.3% with midwife-led care versus 18.9% with obstetrician-led care (ARD=12.0; 95% CI: 10.6–12.8), and cesarean section was 5.7% versus 5.0% (ARD= -0.7; 95% CI: -1.7–0.4). There were 31.6% multiparous women with history of cesarean section that did not have a spontaneous vaginal delivery in the collaborative model compared to 61.8% in the obstetrician-led model (ARD=30.2; 95% CI: 3.7–56.7). For these women, it was differentiated whether childbirth was vaginally assisted or if it was a cesarean section^29^. The study of de Jonge et al.^30^ only compared operative births that included vacuum extraction and forceps delivery. They showed that 24.0% nulliparous women in midwife-led care at the onset of labor experienced an operative birth compared to 36.5% of nulliparous women in obstetrician-led care. When looking at multiparous women, the percentage was 2.2% versus 11.5%. Table 3 provides an overview of the results, which are synthesized in Table 4.

**Table 4:** Synthesis of results of the systematic review

*Authors*	*Spontaneous birth*	*Instrumental birth (vaginal)*	*Cesarean section*	*Augmentation of labor*
**Jackson et al.^29^**	↑	↓	↔	
**Gu et al.^31^**	↑	↓	
**Tracy et al.^32^**	↔	↔	↔	
**de Jonge et al.^30^**		↓		↓

Arrows show the comparison of midwife-led care to obstetrician-led care. ↑ Significant higher rates in midwife-led care. ↓ Significant lower rates in midwife-led care. ↔ No significant difference between the two care models.

### Synthesis of the results

There was a trend towards less intervention among women who received midwife-led care (Table 3). Two studies indicated that the incidence of spontaneous birth was higher in the midwife-led group^29,31^, whereas one study found similar chances among the two care models for spontaneous childbirth^32^. Also, two studies showed a decreased risk for operative birth mode if midwife-led care was applied in early labor^30,31^. Yet, the other two studies reported similar risks or odds, respectively, when comparing midwife-led care to obstetrician-led care regarding operative birth mode^29,32^.

## DISCUSSION

The existing evidence on midwife-led care models during early labor and obstetric outcomes is scarce. Only one study focused on the comparison of midwife-led care to obstetrician-led care and its contribution to interventions during early labor. The study of de Jonge et al.^30^ documented increased administration of labor augmentation if the laboring women were in obstetrician-led care. Regarding birth mode, two studies reported a higher incidence of spontaneous birth if midwife-led care was received. Two of the studies documented a lower risk for cesarean section if they received midwife-led care in early labor, whereas two studies did not find a decreased risk for operative childbirth.

There is a high level of interest in understanding the beneficial contribution of midwifery care to perinatal outcomes for mothers and children^20,24^. Yet, this review shows a lack of evidence when focusing on a specific labor stage. Early labor is a time when parents-to-be often have to cope on their own without professional support^12^. Hospital admission is often delayed due to the potential threat of early labor interventions on cost increase and on birth outcomes^1,11^. Only de Jonge et al.^30^ investigated on that matter and found that labor augmentation can be decreased if women receive midwife-led care at onset of labor. Some women have a bigger need for hospital admission early in labor because obstetric interventions such as pharmacological pain management is favoured^4^. In that case, Coates et al.^14^ demonstrated that obstetricians are more open towards medical interventions than midwives. However, due to the lack of sufficient research, it remains unknown if such attitudes are associated with an increased application of obstetric interventions during early labor and how they relate to birth outcomes. Therefore, further research is needed to better understand the contribution of care models to the intervention rate during early labor. Also, a deeper understanding of the correlation between early labor interventions and birth outcomes should be pursued^1^. It remains uncertain whether the timing of the first intervention is relevant regarding obstetric outcomes. Rota et al.^10^ argue that, in particular, the cascade that follows an intervention could be decisive for the final obstetric outcome.

The results regarding care model received during early labor and its influence on birth mode are controversial. Tracy et al.^32^ found no statistically relevant association of care model during early labor and birth mode. Gu et al.^31^ and Jackson et al.^29^ showed that midwife-led care initiated in the early stages of labor was associated with a lower risk of operative delivery and a higher likelihood of spontaneous birth. However, the magnitude and precision of these effects differ considerably. In particular, the analysis by Gu et al.^31^ was associated with wide confidence intervals, likely reflecting the relatively small sample size and indicating substantial uncertainty and limited precision of the effect estimates. Nevertheless, these results are in line with results of various studies that investigated the contribution of midwife-led care and obstetrician-led care to birth mode in general. Sandall et al.^20^ reported a significant increase in spontaneous childbirth and a significant decrease in cesarean section for women in midwife-led care. However, the majority of studies did not differentiate between stages of labor. Only Jackson et al.^29^ clearly differentiated between midwife-led care and obstetrician-led care during early labor and later in labor. They showed similar results regarding birth mode if midwife-led care was provided early in labor or later in labor. Therefore, it remains unknown if care models specifically applied in early labor support optimal birth mode.

The absence of a standardized definition of midwife- and obstetrician-led care makes researching the topic difficult^17,18^. Midwife-led care models are often described within a continuum of care, which does not necessarily reflect the real-world context. In some countries, midwives act in the leading role in providing maternity care only during a specific part of the perinatal period, such as during childbirth^17,18^. The unclear specification of midwife-led care makes it difficult to attribute a decrease in early labor intervention rates and improved birth outcomes to midwives being the lead health professionals in early labor. However, several studies highlighted the fact that birth outcomes are improved and satisfaction with care is increased when continuity of midwifery care is applied^20,24^. A comparison of midwife-led care without a continuum of care versus midwife-led care within a continuum showed a higher probability of spontaneous childbirth, lower administration of epidural anesthesia, vaginal operative births, and fewer complications during childbirth^18^. Also, focus group discussions with primiparous women showed^4^ that women who are cared for by a known midwife have fewer hesitations in calling for professional help when labor is starting. Sometimes advice and guidance through a telephone call can be enough for women to feel safe at home, and therefore, hospital admission is not yet needed^12^. This could imply that continuity of care can be beneficial in delaying hospital admission, which has already been shown to be beneficial regarding health outcomes as well as institutional resources^1,11^. Further research is therefore needed to define different care models and to understand how midwife-led care at the onset of labor influences birth outcomes.

### Strengths and limitations

This systematic review has identified the lack of sufficient research in care models and early labor. It followed a rigorous approach guided by the Cochrane handbook for systematic reviews and interventions^26^. A sensitive search strategy was performed to find all relevant articles on the subject. Yet, a weakness of this review is the low number of initial studies of which only one study focused on labor augmentation. The quality of the included studies was in general good, with a low risk of bias, except for one study^31^ which was found to have some concerns due to the unavailability of a study protocol. However, this protocol was translated by Sandall et al.^20^ who assessed a high risk of bias in the domain ‘reporting of results’. Due to this, the study by Gu et al.^31^ shows a deficit of quality.

The studies showed a large degree of heterogeneity, including in the definition of midwife-led care, the extent to which midwives act in the leading role, and whether care is provided within a continuum. Therefore, it is recommended to work on a general definition of midwife-led care and obstetrician-led care. Heterogeneity was also seen in the various approaches to measuring birth outcomes. For example, de Jonge et al.^30^ analyzed vacuum or forceps delivery as overall operative delivery. In contrast, Gu et al.^31^ described vaginal operative births as spontaneous delivery. Due to the high heterogeneity in intervention description and outcome measures and the modest number of studies included, it was deemed unreasonable to perform a meta-analysis. Therefore, results were only analyzed descriptively. Additionally, potential publication bias should be considered, as relevant unpublished studies may have not been included.

## CONCLUSIONS

There is a lack of knowledge about the contribution of midwifery care during early labor and its impact on early labor interventions and subsequent birth outcomes. More attention should be given to early labor care in order to improve outcomes for laboring women and their partners. Recognizing the potential benefits of midwife-led care during this phase could lead to initiatives aimed at promoting such care across various settings. Further research is essential to define different care models and their contribution to early labor.

## Supplementary Material



## Data Availability

The data supporting this research are available from the authors on reasonable request.
