# Supplementary material for: Midwife-led care versus obstetrician-led care for childbearing women in early labor: A systematic review
Source: Eur J Midwifery. 2026 Jun 15;10:10.18332/ejm/218434. doi: 10.18332/ejm/218434 (PMC13270553; doi:10.18332/ejm/218434)
Supplement: Supplementary file 1 [file EJM-10-20-s1.pdf]

PRISMA 2020 Checklist

Section and Topic	Item #	Checklist item	Location where item is reported
**TITLE**			
Title	1	Identify the report as a systematic review.	Title page
**ABSTRACT**			
Abstract	2	See the PRISMA 2020 for Abstracts checklist.	
**INTRODUCTION**			
Rationale	3	Describe the rationale for the review in the context of existing knowledge.	Page 3-5
Objectives	4	Provide an explicit statement of the objective(s) or question(s) the review addresses.	Page 5
**METHODS**			
Eligibility criteria	5	Specify the inclusion and exclusion criteria for the review and how studies were grouped for the syntheses.	Page 7-9
Information sources	6	Specify all databases, registers, websites, organisations, reference lists and other sources searched or consulted to identify studies. Specify the date when each source was last searched or consulted.	Page 5-7
Search strategy	7	Present the full search strategies for all databases, registers and websites, including any filters and limits used.	Annex 1
Selection process	8	Specify the methods used to decide whether a study met the inclusion criteria of the review, including how many reviewers screened each record and each report retrieved, whether they worked independently, and if applicable, details of automation tools used in the process.	Page 5-9
Data collection process	9	Specify the methods used to collect data from reports, including how many reviewers collected data from each report, whether they worked independently, any processes for obtaining or confirming data from study investigators, and if applicable, details of automation tools used in the process.	Page 8-9
Data items	10a	List and define all outcomes for which data were sought. Specify whether all results that were compatible with each outcome domain in each study were sought (e.g. for all measures, time points, analyses), and if not, the methods used to decide which results to collect.	Page 8, 11
	10b	List and define all other variables for which data were sought (e.g. participant and intervention characteristics, funding sources). Describe any assumptions made about any missing or unclear information.	Page 7
Study risk of bias assessment	11	Specify the methods used to assess risk of bias in the included studies, including details of the tool(s) used, how many reviewers assessed each study and whether they worked independently, and if applicable, details of automation tools used in the process.	Page 9
Effect measures	12	Specify for each outcome the effect measure(s) (e.g. risk ratio, mean difference) used in the synthesis or presentation of results.	Table 4
Synthesis methods	13a	Describe the processes used to decide which studies were eligible for each synthesis (e.g. tabulating the study intervention characteristics and comparing against the planned groups for each synthesis (item #5)).	Page 7-9, Figure 1
	13b	Describe any methods required to prepare the data for presentation or synthesis, such as handling of missing summary statistics, or data conversions.	Page 9
	13c	Describe any methods used to tabulate or visually display results of individual studies and syntheses.	Table 5
	13d	Describe any methods used to synthesize results and provide a rationale for the choice(s). If meta-analysis was performed, describe the model(s), method(s) to identify the presence and extent of statistical heterogeneity, and software package(s) used.	Page 9
	13e	Describe any methods used to explore possible causes of heterogeneity among study results (e.g. subgroup analysis, meta-regression).	NA
	13f	Describe any sensitivity analyses conducted to assess robustness of the synthesized results.	NA
Reporting bias assessment	14	Describe any methods used to assess risk of bias due to missing results in a synthesis (arising from reporting biases).	Page 9
Certainty assessment	15	Describe any methods used to assess certainty (or confidence) in the body of evidence for an outcome.	Page 11-12

PRISMA 2020 Checklist

Section and Topic	Item #	Checklist item	Location where item is reported
**RESULTS**			
Study selection	16a	Describe the results of the search and selection process, from the number of records identified in the search to the number of studies included in the review, ideally using a flow diagram.	Page 10, Figure 1
	16b	Cite studies that might appear to meet the inclusion criteria, but which were excluded, and explain why they were excluded.	NA
Study characteristics	17	Cite each included study and present its characteristics.	Page 11, Table 3
Risk of bias in studies	18	Present assessments of risk of bias for each included study.	Page 11-12, Figure 2
Results of individual studies	19	For all outcomes, present, for each study: (a) summary statistics for each group (where appropriate) and (b) an effect estimate and its precision (e.g. confidence/credible interval), ideally using structured tables or plots.	Page 12-14, Table 4
Results of syntheses	20a	For each synthesis, briefly summarise the characteristics and risk of bias among contributing studies.	Page 11-12, Table 3
	20b	Present results of all statistical syntheses conducted. If meta-analysis was done, present for each the summary estimate and its precision (e.g. confidence/credible interval) and measures of statistical heterogeneity. If comparing groups, describe the direction of the effect.	Page 14-15
	20c	Present results of all investigations of possible causes of heterogeneity among study results.	Page 12-14
	20d	Present results of all sensitivity analyses conducted to assess the robustness of the synthesized results.	NA
Reporting biases	21	Present assessments of risk of bias due to missing results (arising from reporting biases) for each synthesis assessed.	Page 11-12
Certainty of evidence	22	Present assessments of certainty (or confidence) in the body of evidence for each outcome assessed.	Page 11-12
**DISCUSSION**			
Discussion	23a	Provide a general interpretation of the results in the context of other evidence.	Page 15-17
	23b	Discuss any limitations of the evidence included in the review.	Page 17-18
	23c	Discuss any limitations of the review processes used.	Page 17-18
	23d	Discuss implications of the results for practice, policy, and future research.	Page 18
**OTHER INFORMATION**			
Registration and protocol	24a	Provide registration information for the review, including register name and registration number, or state that the review was not registered.	Page 22
	24b	Indicate where the review protocol can be accessed, or state that a protocol was not prepared.	Page 22
	24c	Describe and explain any amendments to information provided at registration or in the protocol.	NA
Support	25	Describe sources of financial or non-financial support for the review, and the role of the funders or sponsors in the review.	Page 22
Competing interests	26	Declare any competing interests of review authors.	Page 22
Availability of data, code and other materials	27	Report which of the following are publicly available and where they can be found: template data collection forms; data extracted from included studies; data used for all analyses; analytic code; any other materials used in the review.	Page 22

From: Page MJ, McKenzie JE, Bossuyt PM, Boutron I, Hoffmann TC, Mulrow CD, et al. The PRISMA 2020 statement: an updated guideline for reporting systematic reviews. BMJ 2021;372:n71. doi: 10.1136/bmj.n71

Annex 1 – Search strategy for systematic review

Table 6: Search string of all included databases for the systematic review

#	Search string	Results
**Pubmed**		
**11**	((((((((((expecting) OR (expectant)) OR (parous)) OR (parturient)) OR (childbearing)) AND (((((woman) OR (women)) OR (mother*)) OR (person)) OR (people))) OR ("parent to be")) OR ((pregnant women[MeSH Terms]) OR (mothers[MeSH Terms]))) AND (((((((midwife-led care) OR (midwifery-led care)) OR (midwife-led continuity care)) OR (midwifery-led continuity care)) OR (caseload midwife*)) OR (caseload midwives)) OR (midwifery care)) OR (midwife*))) AND ((((("standard care") OR (obstetrician-led care)) OR ("medically-led care")) OR (obstetrician*)) OR (standard of care[MeSH Terms]))) AND ((((labour) OR (labor)) AND (((augmentation) OR (stimulation)) OR (duration))) OR ((((((((((((analgesia) OR (pain management)) OR (anaesthesia)) OR (tocolysis)) OR (tocolytic rest)) OR (pharmacologic rest)) OR (satisfaction)) OR ("satisfaction with care")) OR (birth mode)) OR ("mode of birth")) OR (intervention*)) OR (early medical intervention[MeSH Terms])) OR (natural childbirth[MeSH Terms])))) AND (((((((((latent phase) OR (latent stage)) OR (first stage)) AND ((labor) OR (labour)))) OR ("early labour")) OR ("early labor")) OR (labor onset[MeSH Terms])) OR ((((((childbirth) OR (labor)) OR (labour)) OR (birth)) OR (labor, obstetric[MeSH Terms])) OR (parturition[MeSH Terms])))	160
	OR	**369**<br><br>**Update: 48**
**12**	(((((((((expecting) OR (expectant)) OR (parous)) OR (parturient)) OR (childbearing)) AND (((((woman) OR (women)) OR (mother*)) OR (person)) OR (people))) OR ("parent to be")) OR ((pregnant women[MeSH Terms]) OR (mothers[MeSH Terms]))) AND ((((((((midwife-led care) OR (midwifery-led care)) OR (midwife-led continuity care)) OR (midwifery-led continuity care)) OR (caseload midwife*)) OR (caseload midwives)) OR (midwifery care)) OR (midwife*)) OR ((((("standard care") OR (obstetrician-led care)) OR ("medically-led care")) OR (obstetrician*)) OR (standard of care[MeSH Terms])))) AND ((((((((latent phase) OR (latent stage)) OR (first stage)) AND ((labor) OR (labour)))) OR ("early labour")) OR ("early labor")) OR (labor onset[MeSH Terms]))	220
**CINAHL**		
**8**	(((pregnant OR expecting OR expectant OR parous OR parturient OR childbearing) AND (wom?n OR mother* OR person OR people)) OR "parent to be" OR MW expectant mothers) AND (((midwife-led OR midwifery-led OR midwif*) AND (care OR continuity of care)) OR (caseload midwif* OR caseload midwives)) AND (((obstetrician OR obstetrician-led OR medically-led OR standard OR conventional OR usual OR conventional) AND (care OR treatment)) OR MW standard of care) AND ((labo#r AND (augmentation OR stimulation OR duration)) OR (analgesia or pain relief or pain management or pain control or pain medication OR anaesthesia OR tocolysis OR tocolytic rest OR pharmacologic rest OR satisfaction* OR birth mode OR mode of birth OR)) AND (((childbirth OR birth OR labo#r) OR MW labor) OR ((labo#r onset or latent stage of labo#r or onset of labo#r or latent phase of labo#r or early labo#r) OR MW labor stage first))	113
	OR	214, **211** available<br><br>**Update: 21**
**12**	(((pregnant OR expecting OR expectant OR parous OR parturient OR childbearing) AND (wom?n OR mother* OR person OR people)) OR "parent to be" OR MW expectant mothers) AND ((((midwife-led OR midwifery-led OR midwif*) AND (care OR continuity of care)) OR (caseload midwif* OR caseload midwives)) OR (((obstetrician OR obstetrician-led OR medically-led OR standard OR conventional OR usual OR conventional) AND (care OR treatment)) OR MW standard of care)) AND ((labo#r onset or latent stage of labo#r or onset of labo#r or latent phase of labo#r or early labo#r) OR MW labor stage first)	111

Web of Science		
**8**	(ALL=(pregnant OR expectant OR expecting OR parous OR parturient OR childbearing)) AND ALL=(wom?n OR mother* OR person OR people) AND ((ALL=(midwif* OR midwife-led OR midwifery-led)) AND ALL=(care OR continuity care)) OR ALL=(caseload midwif* OR caseload midwives) AND (ALL=(standard OR obstetrician* OR obstetrician-led OR usual OR conventional)) AND ALL=(care OR treatment) AND ((ALL=(labo$r)) AND ALL=(augmentation OR stimulation OR duration)) OR ALL=(intervention* OR analgesia OR pain management OR anaesthesia OR tocolysis OR tocolytic rest OR pharmacologic rest OR satisfaction* OR birth mode AND (ALL=(childbirth OR birth OR lab$r) OR ALL=(latent phase lab$r OR latent stage lab$r OR labo$r onset OR early lab$r))	291
	OR	559
**12**	(ALL=(pregnant OR expectant OR expecting OR parous OR parturient OR childbearing)) AND ALL=(wom?n OR mother* OR person OR people) AND (((ALL=(midwif* OR midwife-led OR midwifery-led)) AND ALL=(care OR continuity care)) OR ALL=(caseload midwif* OR caseload midwives) OR (ALL=(standard OR obstetrician* OR obstetrician-led OR usual OR conventional)) AND ALL=(care OR treatment)) AND ALL=(latent phase lab$r OR latent stage lab$r OR labo$r onset OR early lab$r)	**288**<br>**Update: 27**
**Cochrane library**		
**9**	((pregnant OR expecting OR expectant OR parous OR parturient OR childbearing) AND (wom?n OR mother* OR person OR people OR parent*)) AND (((midwife-led OR midwif* OR midwifery-led) AND (care OR care continuity)) OR (caseload midwif* OR caseload midwives)):ti,ab,kw AND (((standard OR usual OR obstetrician OR obstetrician-led OR medically-led) AND (care)) OR (standard of care)):ti,ab,kw AND (((labor OR labour) AND (augmentation OR stimulation OR duration)) OR (intervention* OR analgesia OR pain management OR anaesthesia OR tocolysis OR tocolytic rest OR pharmacologic rest OR satisfaction OR birth mode) OR (early medical intervention)):ti,ab,kw AND (((childbirth OR labor OR labour OR birth) OR (labor, obstetric)) OR ((latent stage OR latent phase OR early OR onset) AND (labor OR labour)))	**227**<br>**Update: 0**
**10**	((pregnant OR expecting OR expectant OR parous OR parturient OR childbearing) AND (wom?n OR mother* OR person OR people OR parent*)) AND ((((midwife-led OR midwif* OR midwifery-led) AND (care OR care continuity)) OR (caseload midwif* OR caseload midwives)) OR (((standard OR usual OR obstetrician OR obstetrician-led OR medically-led) AND (care)) OR (standard of care))) AND ((latent stage OR latent phase OR early OR onset) AND (labor OR labour))	459

First search conducted in January 2024, updated in June 2024. Results highlighted in grey.

© 2026 Mueller A.N. et al.
